# Implementing new care models: learning from the Greater Manchester demonstrator pilot experience

**DOI:** 10.1186/s12875-018-0773-y

**Published:** 2018-06-19

**Authors:** Rebecca Elvey, Simon Bailey, Kath Checkland, Anne McBride, Stephen Parkin, Katy Rothwell, Damian Hodgson

**Affiliations:** 1 The University of Manchester, Oxford Road, Manchester, M13 9PL UK; 2 Radcliffe Observatory Quarter, University of Oxford, Woodstock Road, Oxford, OX2 6GG UK; 3 NIHR Collaboration for Leadership in Applied Health Research and Care (CLAHRC), Salford Royal Foundation Trust, Stott Lane, Salford, M6 8HD UK

**Keywords:** General practice, Qualitative research, Primary health care, Health services evaluation, Organizational innovation, Access to health care

## Abstract

**Background:**

Current health policy focuses on improving accessibility, increasing integration and shifting resources from hospitals to community and primary care. Initiatives aimed at achieving these policy aims have supported the implementation of various ‘new models of care’, including general practice offering ‘additional availability’ appointments during evenings and at weekends. In Greater Manchester, six ‘demonstrator sites’ were funded: four sites delivered additional availability appointments, other services included case management and rapid response. The aim of this paper is to explore the factors influencing the implementation of services within a programme designed to improve access to primary care. The paper consists of a qualitative process evaluation undertaken within provider organisations, including general practices, hospitals and care homes.

**Methods:**

Semi-structured interviews, with the data subjected to thematic analysis.

**Results:**

Ninety-one people participated in interviews. Six key factors were identified as important for the establishment and running of the demonstrators: information technology; information governance; workforce and organisational development; communications and engagement; supporting infrastructure; federations and alliances. These factors brought to light challenges in the attempt to provide new or modify existing services. Underpinning all factors was the issue of trust; there was consensus amongst our participants that trusting relationships, particularly between general practices, were vital for collaboration. It was also crucial that general practices trusted in the integrity of anyone external who was to work with the practice, particularly if they were to access data on the practice computer system. A dialogical approach was required, which enabled staff to see themselves as active rather than passive participants.

**Conclusions:**

The research highlights various challenges presented by the context within which extended access is implemented. Trust was the fundamental underlying issue; there was consensus amongst participants that trusting relationships were vital for effective collaboration in primary care.

## Background

Demographic shifts, changes in economic conditions and increased demand on services have brought new urgency to the question of how best to organise health care services. In meeting these challenges, United Kingdom (UK) health policy since the year 2000 has consistently emphasised improving accessibility [1], increasing integration [1, 2] and shifting resources from hospital to community and primary care [3]. Resources have been provided for several initiatives aimed at increasing integrated working practices at the organisational and service levels of health and social care [4–7]. The latest government vision is of an’ integrated’,’ accessible’ health service, with primary care playing a key role in developing ‘locally led’, ‘innovative’ services or ‘new care models’ [8]. Several initiatives, including the Vanguard programme [9] and the Prime Minister’s GP Access fund [10] have been funded and through these, sites are providing services using various care models [10] including GP practices offering ‘additional availability’ appointments during evenings and at weekends.

In Greater Manchester, the local health and social care strategy set out ambitions to transform primary care, focussing particularly on providing accessible and responsive services [11], and also redesigning and integrating primary and secondary care [12]. In June 2013, the National Health Service (NHS) England Local Area Team invited general practices and clinical commissioning groups (CCGs) in Greater Manchester to submit proposals for a ‘demonstrator pilot’ programme (the demonstrators), informed by the Greater Manchester primary care strategy [13]. The specifications were that they must: support a defined community of at least 30,000 people; support the delivery of integrated services across primary, community and social care; consider the use of innovative or enhanced technology; and extend access to primary care. The aim of the programme was to identify issues and test solutions in practice to inform the Greater Manchester primary care strategy going forward. Eighteen applications were received and six ‘demonstrator sites’ were funded: four sites provided additional availability of appointments in general practice, other services included case management and rapid response. Although the short timeline for submission of proposals encouraged applicants to make use of existing relationships, many proposals included plans to forge new collaborations between general practices in an area, often with the encouragement and support of the relevant CCG. Table 1 summarises the services provided.

**Table 1:** TIDieR Checklist description of the demonstrator pilot sites

Item number	Item
1	BRIEF NAME Site A: Pro-active case management for care home residents
2	WHY High ambulance and GP callouts to care homes; too many non-elective admissions to hospital; Care home residents having long lengths of stay in hospital; lack of access to clinical (GP) records from care homes. Goals: to improve access to clinical care outside hospital, specifically reducing GP and ambulance call-outs, hospital attendances and admissions, to facilitate integrated records and allow direct patient access to these.
3 4	WHAT Pro-active case management for adult residents, most aged 65+, of five care homes, registered with one of three general practices in one CCG area. Risk-stratifying care home residents and providing enhanced care planning, including end of life and crisis planning, using risk stratification. Procedure: Care home residents were risk stratified, using the model previously employed in Greater Lever. For each, the case manager, carried out an initial, face-to-face holistic assessment and put a care plan in place, which was recorded on the GP system using a template. At the start of the demonstrator the case manager had access to general practice records via a computer in her office, partway through the demonstrator, she acquired direct read-write access to the records via a laptop. After the initial assessment, the case manager would manage patients using a video conferencing facility. Materials: General practices involved used EMIS^a^, Vision^a^ and TPP^a^, laptop provided to the case manager.
5	WHO The service was provided by an advanced nurse practitioner (ANP) who worked during the demonstrator as a case manager (seconded from an acute trust), with input from care home carers and managers, GPs, practice managers and other administrative staff, a CCG pharmacist and members of the local Mental Health Trust’s dementia team. CCG and CSU project managers and the integrated care lead (from the local Foundation Trust) also contributed.
6	HOW An initial face to face assessment, followed by case management of patients using a video conferencing facility, with the ANP ‘ringing in’ to run through the residents on her caseload with care home staff, hearing about any changes and performing consultations with patients, where necessary.
7	WHERE The assessments and consultations took place in care homes, additional work was undertaken in general practices.
8	WHEN AND HOW MUCH Each care home resident had one initial assessment and then consultations were performed as required.
9	TAILORING Individual assessments were undertaken and care plans produced for each care home resident receiving case management.
10	MODIFICATIONS In practice, the video conferencing technology was not used for both technical and organisational reasons. Rather, the care home staff contacted the ANP by telephone, to discuss residents or to ask her to visit the home. Notably, when at a home, the ANP was often asked, by care home staff, to respond to acute problems for residents that were not on her caseload.
Item number	Item
1	BRIEF NAME Site B: Additional availability appointments
2	WHY Difficulty for patients in obtaining timely and convenient access to general practice; too many emergency hospital admissions. Goals: To improve access to care, specifically providing quicker and more convenient access to routine primary care, reducing attendances at A&E.
3 4	WHAT Additional availability appointments for registered patients of five practices in a township plus one other three miles away, (c. 33,000) Procedure: Additional availability appointments were provided by two GPs, working 6.30 pm to 8 pm Monday to Friday and 8 am to 6 pm at weekends. Three of the practices involved were housed within a purpose - built primary care centre; two of these practices and the practice located outside Radcliffe were owned by the same GP partner. Most appointments were pre-booked, with six kept as emergency appointments for allocation after 6 pm. From 6 pm the practice phone lines diverted to A Healthier Radcliffe. Materials: The appointment booking system was hosted at one practice and the other five logged into this to book appointments. All six practices used Vision with access to the full record, allowed through a data sharing agreement on a read-write basis. GPs used a smartcard to log into each practice system.
5	WHO Two GPs and receptionists.
6	HOW Face to face appointments.
7	WHERE GP practices.
8	WHEN AND HOW MUCH Patients booked appointments as required. Each appointment was 10 min in length, 18 appointments per day were provided Monday –Friday and 12 per day Saturday and Sunday.
9	TAILORING N/A
10	MODIFICATIONS N/A
Item number	Item
1	BRIEF NAME Site C: Additional availability appointments; responsiveness appointments; homelessness service; extension of specialist advice lines
2	WHY Some patients being unable to access timely GP appointments; patients with long term conditions not having timely access to a healthcare professional; insufficient healthcare provision for homeless people. Goals: To improve access to care, specifically reducing A&E attendances, by providing urgent same day (responsiveness) and additional availability appointments in general practice. To improve specialist primary care services and reduce secondary care planned activity, by shifting specialist service provision from secondary to primary care.
3	WHAT 1. Additional availability appointments (33/35 practices). Procedure: Additional availability appointments were provided at four ‘host’ practices. The additional availability appointments were provided by 1 GP at each site, between 6 pm and 8 pm Monday to Friday, and 9 am to 11 am at weekends. The participating practices across the CCG area and A&E at the local acute trust booked appointments at the host practice, which were available on a quota basis, until 1 pm and then made available on a first come first served basis. Materials: Practices logged into the host practice’s system. All practices ran EMIS, either EMIS web or as streaming practices with access to the full record, allowed through a data sharing agreement, on a read-only basis.
4	2. Responsiveness appointments (31/35 practices); Procedure: Practices fitted the responsiveness appointments within the regular working hours of the practice. For example, one practice allocated four telephone triage slots and four appointments to the on – call doctor and two appointments each to all other doctors (the number of doctors in the practice varied). Materials: a macro was put onto each practice system and used to log the outcome of appointment. 3. Homelessness service (1 practice); Procedure: provided under a Locally Enhanced Service arrangement, run at a practice with a large local homeless population (often transient). A health questionnaire for patients was completed, to ascertain health needs and then the patient was signposted to various services (clinics for dressings, immunisations, substance misuse services), several of which operated from the same premises as the practice. 4. Extension of specialist advice lines; Procedure: The ‘specialist advice lines’ were a facility for GPs to get advice from hospital consultants. The service was pre-existing and the additional specialities were added as part of the demonstrator. Materials: Advice lines operated through a dedicated email address for GPs to use.
5	WHO The additional availability appointments were provided by 1 GP at each site, supported by two reception staff, Lead organisation was a GP federation; some additional availability appointments were staffed by locum GPs; the A&E department could refer into the additional availability appointments, local voluntary services could refer into the homelessness service. Hospital consultants staffed the advice lines.
6	HOW Appointments took place face to face and via the telephone.
7	WHERE GP practices.
8	WHEN AND HOW MUCH Patients booked appointments as required. Each additional availability appointment was 10 min in length, 12 appointments per day were provided Monday-Sunday.
9	TAILORING N/A
10	MODIFICATIONS Some changes to the original timings and booking arrangements were made. The weekday additional availability appointments were originally offered entirely on a quota basis and the weekend appointments continued until 12 pm. It appeared some GPs ended up seeing the patient again in normal surgery hours after the additional availability appointment, because they were unsure about what had happened at the appointment. Some practices did not participate in providing the responsiveness appointments; Reasons for non - participation included a lack of capacity in the practice for responsiveness, concerns around IG for one practice and proximity, and being situated on the CCG geographical border.
Item number	Item
1	BRIEF NAME Site D: Additional availability appointments; GP-led care planning; multi-skilled care worker led care planning; hospital navigator service
2	WHY Too much demand on general practice; ‘inappropriate’ use of A&E for problems that could be handled in general practice; A&E used by frail elderly that resulted in avoidable admissions; increase in A&E attendances from 1 pm onwards (when practices are open). Goals: To develop integrated care in line with the CCG strategy. To improve access to care, specifically access to general practice, reduced A&E attendances and hospital admissions. To improve care of the frail elderly through care planning. To develop the IT infrastructure, specifically to allow hub clinicians to access patients’ records, allow practices and patients to book appointments at the hub (a GP practice), and let practices know when their patients are in hospital.
3	WHAT 1. Additional availability appointments for patients registered with GPs in one locality. Procedure: A hub was set up to provide additional GP and nurse appointments, with three nurse clinics and three GP sessions each day. GPs provided additional appointments 4 pm to 9 pm on weekdays and 10 am to 8 pm at weekends. Practices ran the appointment bookings until 6 pm, after which time patients could phone and book directly. The acute trust provided a late-night path lab collection. Materials: Four practices used EMIS, two used Vision. Host practice accessed summary care record on Adastra* on a read-only basis. 2. Care planning Procedure: GPs produced care plans for their frailest elderly patients. The multi-skilled care worker visited patients aged 85 and over at home to identify and assess their needs and produce a care plan.
4	3. Navigator service Procedure: The navigator kept track of presentations to one local A&E department, focussing particularly on those aged 65 and over, so tended to see patients with confusion, falls, and long term conditions, particularly multiple sclerosis and chronic obstructive pulmonary disease. She assessed each patient (each patient was also assessed by the medical team and had tests done as appropriate). Where patients were medically fit and did not need to be admitted, the navigator took responsibility for ensuring that the relevant support was in place, either in the form of a placement, if they were not safe to return home, or home support services (e.g. from team providing crisis response).
5	WHO The project lead was a GP. Local out of hours provider (supplied GPs and receptionists for additional availability appointments); the navigator was an occupational therapist based at a local general hospital, the multi-skilled care worker was based at a foundation trust.
6	HOW See ‘procedure’ for a description of how each component operated.
7	WHERE Additional availability appointments took place in person, at GP practices, care planning took places in GP practices and at patients’ homes, the navigator service operated in hospital.
8	WHEN AND HOW MUCH Patients booked appointments as required. Each additional availability appointment was 15 min in length, 28 appointments per day were provided Monday-Friday, 51 on Saturday and 24 on Sunday.
9	TAILORING Care plans were prepared for individual patients. The navigator service arranged tailored care packages for patients.
10	MODIFICATIONS The additional availability GP appointments were typically booked, but the nursing ones were less popular and were replaced with GP appointments after six weeks. Issues arose as practices which had been allocated appointments were unwilling to give up their allocated slots to other practices which had filled theirs.
Item number	Item
1	BRIEF NAME Site E: Additional availability appointments; mental health crisis clinics.
2	WHY Too much demand on general practice; lack of an accessible mental health service locally. Goals: To improve access to care, specifically providing quicker and more convenient access to routine primary care, reducing attendances at A&E and increasing access to mental health services, by extending access to routine primary care and providing additional mental health services in the community. To make better use of local resources and support the local population to do this, specifically to reduce attendances at A&E, reduce hospital admissions and facilitate quicker discharge from hospital, by providing signposting and education to local services in the community, improving patient pathways and supporting collaboration between professionals in different agencies.
3	WHAT 1. Additional availability appointments for patients registered with GPs in one locality. Procedure: The general practice additional availability appointments ran from the lead practice. A purposely developed Care Diary was used by GPs, the local out of hours provider and A&E staff to book patients into the additional availability appointments. Patients were triaged at A&E and, if the ailment could be managed in the community, they could be booked into a GP or nurse appointment by staff at A&E using the Care Diary. Materials: six practices used EMIS, two used Vision. EMIS practices were able to share records on a read-only basis, Vision practices were not able to access records. Since Dec 2014 all practices have been EMIS web allowing all to share records on a read-only basis.
4	2. Mental health crisis clinics for patients registered with GPs in one locality. Procedure: The clinics were organised by a trained counsellor, who co-ordinated the service and provided appointments, plus other counsellors (and trainees) who also worked at another local general practice. Appointments were provided between 6.30 pm and 9.30 pm, Monday to Friday.
5	WHO The project lead was a GP. The additional availability appointments were provided to registered patients, at the lead practice, by GPs, supported by receptionists, all supplied by the local Out of Hours provider. The mental health appointments were provided by trained counsellors and counselling students. The demonstrator appointed a dedicated project manager partway through. CSU and EMIS also contributed to the project.
6	HOW See ‘procedure’ for a description of how each component operated.
7	WHERE In person, at GP practices,
8	WHEN AND HOW MUCH Patients booked appointments as required. The additional availability appointments were each 10 min in length and 18 appointments were provided per day, Monday-Sunday. The mental health appointments were each one hour in length and three per day were provided, Monday- Friday.
9	TAILORING
10	MODIFICATIONS The additional availability GP appointments were typically booked, but the nursing ones were less popular. Issues arose as practices which had been allocated appointments were unwilling to give up their allocated slots to other practices which had filled theirs. Some local GP practices did not refer patients to the mental health appointments, the lead GP was aware of this but the reasons for non-engagement are not known.
Item number	Item
1	BRIEF NAME Site F: Rapid response step-up service; complex care service; enhanced end of life service; carer needs assessment service; mental health liaison, care homes; end of life training, care homes and locality.
2	WHY Too many non-elective hospital admissions; too many patients dying in hospital; district nurses were under pressure and did not have enough time to provide the right end of life care and support to patients and carers. Goals: To proactively identify and manage people with complex needs via a core integrated team that can draw on specialist support when necessary. To support people with heart failure by extending telehealth services. Support for people to be maintained in their own home or care home where this is their preferred place prior to and including death. A reduction in unplanned, avoidable non-elective activity prior to and including death.
3	WHAT Overall: The demonstrator was part of the restructuring across health and social care, through the development of an ‘integrated hub’ in each CCG locality. The demonstrator took place in one locality, where the first hub had been established. The hub premises accommodated social workers and third sector staff. Stockport had shared patient information via the Stockport Health record which enabled GPs, secondary care and Out of Hours services to access each other’s systems. An extension of the Stockport Health Record, to include health and social care data and integrated care plans, was planned to support the implementation of the Stockport One Integrated Care Team and was further developed within the demonstrator community demonstrator to ensure that the whole range of services within the hub had appropriate access to information. In terms of specific systems operating locally, social care used CareFirst, district nurses used DominiC, the REaCH service used Staffplan, and domiciliary workers users used CM2000 (to log each visit).
4	1. Rapid response step-up service provided to people aged 18 and over. Procedure: GPs referred into the service via a dedicated number at a contact centre when they felt a patient did not need to go to hospital, but needed support putting rapidly in place. Once the GP had made the referral, the patient received a response within 2 h from a team comprising a district nurse and a social worker. The patient could be maintained in their own home or go into a step-up bed. This service ran from 9 am to 5 pm and the intermediate care service provided an Out of Hours service. Materials: six practices used EMIS, two used Vision. EMIS practices were able to share records on a read-only basis, Vision practices were not able to access records. Since Dec 2014 all practices have been EMIS web allowing all to share records on a read-only basis. 2. Complex care service Procedure: the population was risk stratified. Multidisciplinary teams (MDTs), involving a GP and a practice nurse, worked to agree an integrated pathway and model of care for individual patients. The work undertaken followed the same basis as the GP care plans which had already been developed, but allowed other healthcare professionals to contribute to these. The task of coordinating the care plan was undertaken by various professionals (GPs, district nurses, social workers) and also voluntary sector workers. The multidisciplinary group (MDG) was a wider network of professionals which operated at a more strategic level, looking across the locality and identifying, for example, high rates of chronic obstructive pulmonary disease and considering what action should be taken, rather than necessarily focussing only on patients within the high risk stratification. Materials: the People at Risk of Readmission tool was used for risk stratification 3. End of life care service Procedure: The end of life care service was newly designed service that focussed on integrating health and social care. This is a jointly delivered service between district nursing (health) and assistant practitioners (social care) in the community. The service delivers end of life care to people in the last weeks and days of life undertaking joint assessments, care planning and visiting the person in their home to deliver interventions that meet the needs of the patient and their carers or family. The health and wellbeing service was planned as an extension of the existing service, into a different area. The end of life training consisted of delivering a module to care home staff. The dementia-focussed training consisted of several one-hour training sessions delivered to care home staff. Materials: End of life training based on the Six Steps programme and providing follow up telephone support. 4. The mental health liaison in-reach service involved working with three care homes to provide advice and support, particularly care planning.
5	WHO The demonstrator was part of a programme of work developed by the CCG and local authority, a hub was established and a hub co-ordinator was employed; the local Foundation Trust, Community mental health trust and local authority reablement service were involved; Project managers and general practice staff contributed. The MDT and MDGs were comprised of GPs, district nurses, social workers, primary care pharmacist and third sector staff. The end of life service was provided by assistant practitioners (domiciliary workers) from the REaCH service. The end of life training for care homes was provided by end of life facilitators. The health and wellbeing service was led by project managers, liaising with general practice staff. The carer assessments were carried out in general practice, with input from GPs and administrative staff. The mental health liaison in-reach service was provided by a community psychiatric nurse and a support worker.
6	HOW All services were provided in person.
7	WHERE At GP practices, in patients’ homes, in step-up facilities, care homes.
8	WHEN AND HOW MUCH Services were provided to patients as required.
9	TAILORING
10	MODIFICATIONS The aim was for district nurses to be co-located at the hub but this was not possible within the timeframes associated with the demonstrator. In practice, social workers were ‘paperless’ whilst district nurses used paper records.

^a^computer systems used in general practices and/or the companies that supply these systems

Given the rapidity and scale at which new models of service are spreading, evidence about early adopters, such as those in Greater Manchester, is important. There is a growing body of literature about the adoption of health service innovations more generally [14–17], but a relative paucity of high quality research focusing upon the implementation of organisational change in primary care [18]. Furthermore, whilst the importance of context and inter-group relations in supporting or preventing organisational change is well known [19, 20], the primary care context is less well understood [21, 22].

We conducted a process evaluation of the Greater Manchester primary care ‘demonstrator pilot’ programme, designed to identify key learning points from the implementation process to inform future service innovations of this kind. The overall study findings, including a quantitative evaluation, are reported elsewhere [23, 24]. The aim of this paper is to explore in depth the factors influencing the implementation of services within the demonstrator sites, in particular the context in which the services developed and the experiences of people involved with providing them.

## Methods

The qualitative process evaluation used semi-structured interviews. The sampling strategy was purposive in that it was driven by the characteristics of the case study sites and designed to capture a range of views from people working at strategic and operational levels, within the main provider organisations – general practices – and other organisations involved, including hospitals and care homes. This resulted in an evolving list of potential interviewees, as early interviews provided detail on others involved in project delivery, who were then targeted for interview. Potential participants were contacted by email and/or telephone and were supplied with information about the purpose and aims of the study; participants were encouraged to ask questions about the study and the research team prior to interviews. The interviews were conducted in three stages: firstly, interviews with ‘key informants’, typically site strategic leads; second, interviews with a range of people working in various roles at different levels; third, a set of six ‘round up’ interviews close to the end of the demonstrator period, with site strategic leads and a clinician with a key role, designed to gain a retrospective account of each demonstrator.

Semi-structured interview topic guides were devised, which included open-ended questions about the project, its aims and the context in which it had developed, the participants’ own role, experiences of working in the pilot and views about what was working well, what was not working well and possible reasons why. The interviews were audio recorded, with participants’ written consent and transcribed verbatim. Field notes were made during and after interviews. The software package NVivo10 was used to store and manage the data.

SB, RE and DH carried out the interviews between January 2013 and October 2014. All interviewees were experienced and trained in qualitative research to PhD level. Most interviews were conducted in person at the participants’ place of work and four were conducted via telephone. The majority of interviews were individual, with a few conducted in pairs or trios. The interviews lasted between 15 min and 2 h, most being around 1 h long.

Data analysis followed ta thematic approach. Categories were generated through reading the transcripts, with some themes following the research questions, whilst others were derived from the data themselves [25]. Team members read the transcripts and discussed emerging themes. Initial open coding was refined by consensus within the team as a whole, with the agreed coding framework applied to the complete dataset by SP, SB and RE. The process of coding and organising the data was iterative, with the framework being revised and refined as further coding was undertaken and categories and themes grouped together. Feedback on emerging findings was provided by the evaluation team at a series of ‘action learning sets’ that were accessible to all involved with the sites; these were supplemented by the dissemination of interim reports, in addition to the final report.

## Results

Ninety-one people participated in interviews (Table 2). Iterative analysis identified six influencing factors: information technology; information governance; workforce and organisational development; communications and engagement; supporting infrastructure; and federations and alliances. We explore each of these in turn and show the range of views expressed.

**Table 2:** interviewees by demonstrator and role^a^

Demonstrator	Role						
	Manager	Nurse	Doctor	Pharmacist	Support worker	Administrator/other^b^	total
A	7	1	2	1	1	1	13
B	13	0	5	1	1	0	20
C	7	0	3	0	0	1	11
D	9	1	4	0	1	1	16
E	6	1	6	0	0	2	15
F	18	1	1	1	5	0	26
Total	60	4	21	3	5	5	98^c^

^a^hybrid roles have been categorised by professional background of individual when they retain a clinical role (3) and by organisational role when they do not (3)

^b^occupational therapist (1) counsellor (1)

^c^8 individuals double-counted due to roles spanning sites D and E (7) and B, D and E (1)

### Information technology

Innovative use of information technology (IT) was a prerequisite for obtaining funding, but all sites experienced IT challenges, often of unexpected complexity. Several participants described learning from initial ‘teething problems’. Thus one pilot lead, acknowledging that technical problems were frustrating, emphasised that they were not insurmountable:

*We’ve got a couple of different solutions, some a bit more clunky than others, but there are solutions there…So I think the technology I’m sure can be pretty easily worked out in a place like (name) anyway. (GP site lead)*

However, at some sites the technical IT problems were far from ‘easy’ to solve, delaying the initiation of services, reducing efficiency and even preventing some service components being initiated at all. Technical problems affected both hardware and software: system failures; difficulties getting remote access to systems or records from mobile devices; and obstructions to equipment installation were all described. Whilst the precise problems varied between sites, there was a common recognition of initial over-optimism about what IT could deliver and subsequent complications, preventing the implementation of some elements of services:

*EMIS had a configure switch where you can just turn up and log into (it). That didn’t work. There were a couple of things we had to do to test it. Once we realised that (it) wasn’t working, we had to stop. (Project manager)*

The interoperability of practice systems presented key challenges. Practices trying to work together to provide additional appointments had expected to be able to access one another’s patient records. Whilst problems might be expected between practices using different computer systems, even those using the same system experienced setbacks. Further work was necessary to standardise the way the system was used, to gain agreement on this and to train staff. Participants expressed frustration with the interim workarounds developed, which were often lengthy and complex without delivering the expected operability:

*But the Vision 360, although there’s a bit of light at the end of the tunnel, is not the all singing and all dancing as we thought it might be…it’s so long winded to get from A to B. (Project manager)*

Beneath this lay more challenging issues of inter-organisational working and relationships, with some interviewees distinguishing between the technical IT solutions and the more difficult integrative work required:

*The IT integration…is not that bad. That’s just complicated easy stuff…It’s the simple hard stuff. And it’s the relational stuff, it’s trusting somebody outside to come in, that you’ve got really no control over…Who is it? What are they doing? What training? You know... So that integration stuff is, I think a learning that we need to take forward from this…you’ve got to have the GPs on board to allow people to come in and do this… (GP site lead)*

All of the demonstrator areas eventually achieved the expected ‘innovative use of IT’, but it was often more difficult than anticipated and participants were disappointed at not being able to depend on the computer systems to work in the ways they had expected. Workarounds were developed, but more sustainable solutions required extended engagement with suppliers and practices. For practices, having trust in anyone who would have access to their systems was crucial.

### Information governance

Whilst linked to IT, information governance (IG) appeared in these narratives as a separate, complex issue. Respondents described unanticipated problems relating to inflexible governance procedures and disparity in governance protocols between organisations. These were described as ‘hurdles’ and ‘organisational hoops.’ Cultural differences were found between different types of organisation and between healthcare sectors. The quotes below reflect embedded perceptions of approaches to information governance in primary and secondary care respectively, with direct implications for implementation processes:

*…the information governance at the hospital is much tighter than it is in general practice and so what we think is reasonable data to see, the hospital are not altogether happy. So they tend to insert much more stringent criteria than we do. (GP site lead)*

*We’re a big organisation, we have very stringent governance procedures…what the demonstrator’s done is put us working with small, independent businesses, and I guess there’s a flexibility [for general practices]... I think they’ve been a bit frustrated in dealing with a fairly bureaucratic system... [Proposed interventions] had lots of IG issues in them…there were processes to go through. We couldn’t just say ‘yeah, fine, we can do that tomorrow’... (Hospital manager)*

A dialogical approach was therefore required in the demonstrator sites to reconcile these perspectives to allow effective collaboration to take place between different organisations, particularly between primary and secondary care.

The human issues of trust and relationships had important implications for IG. The following quote illustrates the need to gain the trust of practices, which here seem to be portrayed as protective or cautious, in order for people outside the practice to access data:

*We’ve got to earn the trust of the GP practices. First of all, to allow read only access. So that’s been a big barrier to break down, because the practice managers were not keen to allow, even, read only access, and to allow people to actually enter data onto it, that’s a step too far at the moment. (Manager)*

Whilst the provision of honorary contracts offered partial solutions to IG issues, more fundamental solutions involved open and early dialogue.?>

### Workforce and organisational development

Several respondents in each of the four sites extending GP opening hours mentioned the challenge of GP capacity, raising issues such as skill shortages, concerns about work-life balance and sustainability:

*…one of the main problems I think is getting the clinical cover ... they may be able to do it for so long but it’s not sustainable on a long term basis (Site lead)*

These pilots aimed to provide additional *routine* appointments, rather than conventional out of hours care. Thus, alongside routine GP appointments, nurse appointments were also offered. However, there was no standard practice nurse training or skillset amongst nurses working at the sites; therefore, they were not necessarily ‘interchangeable’, yet, this was not always considered when allocating nurses to appointment slots:

*…somebody turned up for a diabetic check-up and the nurse who was on couldn’t do it... (GP)*

A broader issue relating to substitutability (exchanging one type of worker for another) was highlighted by interviewees who described some individuals involved in the demonstrator as being more experienced, or more committed, than the average person in that role. This meant that the role could not be easily replicated. Many demonstrators flourished due to the contribution of individuals who are difficult to replace or replicate, due to their particular competences, ways of working and also the trusting relationships they had established with practices:

*Simply adding six more nurses might not facilitate six times more to be achieved than what [postholder] has done, because she had an established relationship with practices, as well as being a good communicator, so she was trusted and accepted by the practices, so they were happy for her to have access to their systems. But this engagement will not happen automatically: GPs have to feel that they trust people that are doing the work for them. (GP site lead)*

Thus, whilst many people worked hard to support the pilots, rolling out services more widely not only requires enough staff, but also requires understanding of issues of substitutability, working relationships and levels of interpersonal trust established over time.

### Communications and engagement

The demonstrators involved complex networks of organisations; even the site with the smallest population coverage and narrowest focus involved seven different types of organisation. Establishing and maintaining engagement was mentioned by participants at all sites.

Some demonstrators built effectively upon existing relationships. Others, however, carried an over-optimistic expectation that people or organisations that had not worked together previously would immediately do so for the pilot, resulting in delays. Some GP site leads found practice managers and administrators somewhat self-protective when it came to operationalising the plans, reflecting limited trust between some practices:

*The nursing appointments – we could fill them but … [another practice] doesn’t utilise their nurse appointments but won’t let anybody else have them, and you get into this argy-bargy with practice managers then. ‘Well can we use them?’ ‘No, we might use them.’ ‘But you’re not using them’…what I call the general practice little bit of selfishness… they stick to their guns and say ‘these are our appointments and we still want them.’ (GP site lead)*

Personal and direct communication with practices was felt to be vital, with letters to practices setting out plans highlighted as insufficient. Some practice managers reported being asked to carry out extra work at short notice, without having been involved in the planning stages of the project(s), nor their opinion sought. This manager, for example, ascribed ‘teething troubles’ at one site to an unanticipated request to arrange access to a computer system at short notice:

*The first time I was aware (was when people arrived to set up IT access for a nurse practitioner) and (I thought) ‘Oh right’…and that happened on at least three occasions. So three afternoons were completely trashed off…we were messing and mauling about trying to set things up, which irked me a bit, because…I’d not been privy to what this would mean, other than ‘oh she’s [the nurse] going to be able to access (the practice system)’. (Practice manager)*

Sometimes, the demonstrators had acted to foster new relationships, or improve existing ones. For example, two practices providing additional appointments via a collaborative hub had historically tended to work independently. Here, the demonstrator process itself drove more collaborative working between them. Practice managers described how their initial engagement with each other had tended to be task-based, with division of responsibilities and limited discussion. However, their experience of working together led to closer working and managers described starting to telephone each other for advice on day-to-day practice issues not necessarily related to the demonstrator pilots. A strategic lead, who had worked with these practices, expressed a similar view, suggesting that the GPs seemed to have more genuine discussions:

*Well, actually getting (several) practices in a room with two GPs and getting them to agree - that’s not to be underestimated. And when I say agree, I mean agree in the room and agree outside the room. (Site lead)*

Thus, engagement with the project and good communication went hand in hand. When it worked well, those involved felt both informed and consulted, with the ability to influence the pilot. While in some sites, a failure to take a dialogical approach, or to engage, resulted in resistance to changes, ongoing routine interaction through the demonstrators also generated collaborative relations and open, trusting relationships.

### Supporting infrastructure

All demonstrators made use of current local infrastructure, using existing premises that were already equipped. As these were fixed-duration pilots, no demonstrator sought to acquire new premises, although the initiation of one demonstrator coincided with the establishment of a ‘hub’ location. The pilots operated within the wider health and social care infrastructure, with varying amounts of interaction with external organisations. Respondents across all demonstrators referenced local A&E departments, Out-of-Hours providers, the North West Ambulance Service and community pharmacies as important local services. Deciding which support services were needed was a pivotal decision for routine appointments provided during evenings and weekends. For financial and clinical reasons, there was recognition that such support would only entail ‘sufficient’ services and that it would be unfeasible to provide an entire health and social care system around the clock:

*You don’t need a full complement of staff in the evenings and weekends like you do during the day. What you do need is access to enough services to deliver a competent service…path lab stuff, transport and all that sorted out…a district nurse service in the evenings and weekends. (GP site lead)*

Pathology collections during the evening were particularly important for the ‘additional availability’ demonstrators and one site reported positive experiences arranging this with a local hospital. Elsewhere, the lack of infrastructure support, in particular ‘back office’ support functions within the voluntary sector was highlighted. A manager employed by the local authority described some of the practical challenges she had faced when working with voluntary organisations; she seemed to lack confidence in the support available to make systems work together:

*When they want to get onto the systems and get their emails, sometimes it works, sometimes it doesn’t… and we all have a three way conversation to fix this problem in all the four organisations. That in itself is a ridiculously difficult thing to organise, and I’m struggling with it…(Local Authority Manager)*

The time-limited nature of the demonstrators meant that they operated largely within the existing infrastructure, adapting to existing limitations. It was vital therefore that sites considered and planned both the level of necessary support and the level of resource available within external organisations.

### Federations and alliances

Recently, groups of GP practices have started working together in more formal ways, often referred to as GP federations, defined as: groups ‘of practices and primary care teams working together, sharing responsibility for developing and delivering high quality, patient-focussed services for their local communities’ [26]. At two demonstrators, services were running through existing federations. Participants here identified several benefits from working as part of a federation: the ability to combine resources; increased population coverage; enhanced professional standards because of peer support and review; and the fostering of a common identity and purpose amongst practices. One pilot achieved full sharing of patient records, with read-write access between practices; this was singled out as resulting from the formal agreement between the practices in the federation:

*[name] have full access to the patients’ records. That’s the unique bit. (Manager)*

In both areas where demonstrators were run by federations, pre-existing relationships and historical joint working between practices were considered important in supporting the federation establishment. At the site with full shared records, previous joint working was seen as essential to this achievement; collaborators were familiar and trusted:

*GPs are very protective of their patient data…but because the GPs are shareholders of this organisation…they’ve got a vested interest in this organisation, they work collaboratively and they know who they’re sharing their data with…So in order to get that data sharing agreement you have to have some kind of collaboration going on in the background. (Manager)*

As well as these immediate benefits, federations were seen by some as a way to protect primary care in the context of future policy demands, particularly around ‘extended opening hours’ and the associated likely demands on workload:

*I think there’s going to be a…coming together of practices. So going forward I can only see the federation will get bigger as individual practices struggle more to meet all the policy demands…if extended hours becomes the norm. It would be become really hard for an individual practice to do that. (Manager)*

Demonstrator pilots supported by local federations thus benefitted from existing joint working and trust between practices. Notably, these sites found it easier to overcome some of the challenges associated with IT, IG, workforce and communications than other sites, partly through better established relationships and trust and partly through having a formal mechanism to address such issues.

## Discussion

### Summary

This study explored the processes of implementing pilots focussed on access, integration and innovative use of technology in primary care through an evaluation of a programme initiated in Greater Manchester. Our analysis identified six factors which were influential in the implementation of the pilots: information technology; information governance; workforce and organisational development; communications and engagement; supporting infrastructure; and federations and alliances. Underpinning all of these factors was the issue of trust; there was consensus amongst our participants that trusting relationships were vital for collaboration.

### Strengths and limitations

We accessed the experiences of people working at strategic and operational levels within a range of sectors, therefore gaining multiple perspectives on complex and poorly understood processes. Our interviews were mostly undertaken with people engaged with the pilots and therefore, overall, may represent a more ‘interested’ viewpoint. We did not capture patient experiences directly, which is a limitation of this study, although the quantitative component reported elsewhere included an analysis of items from the national GP Patient Survey [23].

### Comparison with existing literature/implications for practice and areas for further research

There are commonalities between our findings and previous studies of implementation in healthcare, which have found factors such as information technology, relationships, communication and organisational culture to be important [7, 14, 16, 19]. Our study confirms these findings, but also extends them. Working relationships have been found to be vital, within and between various health and social care organisations; our data provide examples of the importance of these within general practice and show some of the ways in which these can be fostered. Whilst previous research [14] found relationships between people in senior leadership roles to be important, we found that relationships between practice managers in particular, as well as administrators, were also key. Research also emphasises the importance for change efforts of mobilising valued identities [19]. Disseminating the proposal brief direct to professionals (rather than cascading through commissioners in CCGs) helped to bypass some of the problems associated with resistance to change by professionals [27, 28]. Professionals then took ownership of ‘their’ projects and this helped create the positive and proactive dynamics and trust required to move beyond silos [14, 19]. Although there is a danger that this could also reproduce a narrow set of professional interests [29], the importance of trust to professional enrolment and professional-managerial boundary crossing was key. For example, whilst IT/IG issues were common, what determined whether or not they were surmountable was the extent to which trust existed, or could be developed, between the parties. Our study also provides further detailed evidence about the nature of IT/IG issues in joint working. Going beyond system interoperability (often the goal of integrated care programmes), we have shown that the issues of who can write to your records, who can read them and how decisions about the use of IT get made need careful consideration.

Whilst it is self-evident that communication is important in initiating and sustaining change programmes, our study aligns with existing research which highlights the need for a dialogical approach [30, 31]. By this we mean that it is not enough to simply passively disseminate information; participants need to feel that they are active contributors, understanding what is happening but also able to influence it and change the direction of a project should it be necessary. Some of our participants resented finding out about the projects only when they had to provide help at short notice, a finding which mirrors previous work on implementation in primary care, which found that staff did not feel involved in decision making and that a top-down approach was a negative factor [32, 33]. Furthermore, our data extends the existing research by providing evidence of how primary care implementation is experienced by practice managers. Moreover, our study suggests that, whilst federated working seemed helpful, this seemed to operate via the medium of shared history and the trust which arises out of practical experience of joint working in other spheres. This aligns with findings from research secondary care, which has emphasised the importance of both shared history and a shared purpose [34, 35]. The positive impact of existing federated working may simply reflect this shared history, and we would expect the question of shared purpose to be key to the success of future federations and other similar collaborative ventures.

Whilst we did not use a particular evaluation framework or tool in this study of implementation, there are parallels between our approach and findings and the constructs of the Consolidated Framework for Implementation Research (CFIR), an established framework used in evaluations of implementation [17]. In terms of approach, our focus on context and process fit with the inner and outer settings and process constructs in the CFIR. Our findings on the importance of relationships, communication and engagement in particular mirror the CFIR focus on engaging and involving individuals, networks and communication, culture and compatibility. It may be beneficial to consider the utility of frameworks such as CFIR in the design of future research on organisational change in primary care.

## Conclusions

Taken together, our findings provide some very practical lessons for those seeking to initiate similar cross-boundary projects. Starting small, with trust building over time via the experience of shared working, is likely to be more effective than large scale projects imposed from above. Detailed consideration is also required not just of the mechanics of information technology (‘the complicated easy stuff’) and the legal aspects of information governance, but also issues of ownership, rights to log things to the record and facilitating the development of trust, particularly between general practices. It was crucial that GP practices trusted in the integrity of anyone external, particularly if they were to access data on the practice computer system. The schemes in question found ways to resolve their challenges, through provisional ‘workarounds’ or through more permanent negotiated solutions. This often relied upon the commitment and innovation of individuals within these pilots going beyond their established role both in scope (acting beyond the formal parameters of their role) and in scale (working longer hours). For future initiatives, it is important that all organisations and agencies involved in the design and delivery of innovative models of community-based primary care work to ensure that more suitable time periods for all aspects of project management are provided. Service providers and system leaders, in particular, should consider extended periods of operation, to further enable more sustained and more focused attempts at publicising the service. Having more appropriate time periods in which to plan and operate services would allow greater opportunity for engagement and communication, as well as managing inter/intra organisational expectation. With a clearer understanding of the complex and embedded practical challenges of collaboration developed through this evaluation, practitioners may be better positioned to anticipate and address these challenges. This is of increasing importance as the NHS rushes to implement a range of new models of care [9].

## Abbreviations

A & E: Accident and emergency
CCG: Clinical commissioning group
CSU: Commissioning support unit
EMIS: Egton Medical Information Systems
GP: General Practitioner
IG: Information governance
IT: Information technology
MDG: Multi-disciplinary group
MDT: Multi-disciplinary team
NHS: National Health Service
UK: United Kingdom
